# Transport and Mixing Induced by Beating Cilia in Human Airways

**DOI:** 10.3389/fphys.2018.00161

**Published:** 2018-03-06

**Authors:** Sylvain Chateau, Umberto D'Ortona, Sébastien Poncet, Julien Favier

**Affiliations:** ^1^Aix Marseille Univ, Centre National de la Recherche Scientifique, Centrale Marseille, M2P2, Marseille, France; ^2^Département de Génie Mécanique, Université de Sherbrooke, Sherbrooke, QC, Canada

**Keywords:** mucus, cilia, transport, mixing, pulmonary flow, lattice Boltzmann method, immersed boundary

## Abstract

The fluid transport and mixing induced by beating cilia, present in the bronchial airways, are studied using a coupled lattice Boltzmann—Immersed Boundary solver. This solver allows the simulation of both single and multi-component fluid flows around moving solid boundaries. The cilia are modeled by a set of Lagrangian points, and Immersed Boundary forces are computed onto these points in order to ensure the no-slip velocity conditions between the cilia and the fluids. The cilia are immersed in a two-layer environment: the periciliary layer (PCL) and the mucus above it. The motion of the cilia is prescribed, as well as the phase lag between two cilia in order to obtain a typical collective motion of cilia, known as metachronal waves. The results obtained from a parametric study show that antiplectic metachronal waves are the most efficient regarding the fluid transport. A specific value of phase lag, which generates the larger mucus transport, is identified. The mixing is studied using several populations of tracers initially seeded into the pericilary liquid, in the mucus just above the PCL-mucus interface, and in the mucus far away from the interface. We observe that each zone exhibits different chaotic mixing properties. The larger mixing is obtained in the PCL layer where only a few beating cycles of the cilia are required to obtain a full mixing, while above the interface, the mixing is weaker and takes more time. Almost no mixing is observed within the mucus, and almost all the tracers do not penetrate the PCL layer. Lyapunov exponents are also computed for specific locations to assess how the mixing is performed locally. Two time scales are introduced to allow a comparison between mixing induced by fluid advection and by molecular diffusion. These results are relevant in the context of respiratory flows to investigate the transport of drugs for patients suffering from chronic respiratory diseases.

## 1. Introduction

Computational Fluid Dynamics (CFD) is becoming a powerful tool in the medical context. It provides a good insight of physical phenomena occurring inside the human body without the need of intrusive surgery methods, which often fail to observe the desired phenomenon as they introduce perturbations. Many organs, such as the human heart, have already received a lot of attention from scientists using numerical methods (Khalafvand et al., 2011). However, only few studies focused on modeling the lungs entirely, as it is probably one of the most challenging organ to simulate due to the different length scales involved, from microns for the mucociliary transport to centimeters for the airflow in the upper airways. The transport of mucus depends on its interaction with cilia, whose scale is of the order 10^−6^ m, but is also strongly affected by the numerous bifurcations (length and diameter of order 10^−1^ m in the upper airways) that form the bronchial tree. Some authors have tried to study the entire lung, at the price of severe simplifications: Inagaki et al. (2009) looked at the pressure losses inside the full bronchial system, but neglected the multi-component nature of the flow and the mucociliary transport. Stylianou et al. (2016) looked at the impact of bifurcation for the particle laden flow using Direct Numerical Simulations (DNS), but considered only one bifurcation and did not take into account all the phenomena occurring at the microscale. Given the actual capacities of supercomputers, it is prohibitive to model the entire system while accounting for the multi-component and multi-scale nature of the flow, the deformation of the bronchial tree during a breathing cycle, the heat and mass transfer at the epithelium surface, etc. Hence, many authors restrict their study to a given scale/phenomenon, as it is the case in the present work. Before going any further, it is also worth noticing that, in recent years, the need for efficient methods able to perform the simulation of deformable moving solids in multi-component flows has also been felt in other areas. In this context, the aim of this paper is to present a numerical tool, which can be used to study many biofluidic configurations, such as the transport of nutrients in the brain (Siyahhan et al., 2014), the displacement of ovules in the Fallopian tubes (Anand and Guha, 1978), or even the simulation of industrial micro-mixers (Chen et al., 2013).

In this paper, one considers the mucociliary clearance process (MCC), which is the main defense mechanism developed by the human body to protect itself against foreign particles (like pollutants, allergens, bacteria, etc.) which are inhaled during the breathing process. Its principle is simple: a layer of fluid called Airways Surface Liquid (ASL) covers the surface of the airways. The inhaled particles are deposited onto it, and then transported to the stomach thanks to the combined motion of the cilia tufts that cover the epithelial surface. In the two-phase model adopted here, it is generally assumed that the ASL is in fact the superposition of two different fluid layers: the periciliary liquid (PCL), and the mucus phase above it (Knowles and Boucher, 2002). In this model, the PCL can be viewed as a Newtonian fluid similar to water. However, the modeling of PCL remains an open question in the literature, as its experimental characterization is not yet fully understood. Hence, other models exist such as, for example, the one of Button et al. (2012), where the mucus is depicted as a gel made of reticulated mucins. The interesting proposed idea being that, if the PCL is not thick enough and/or has a low hydration, then the mucus-gel may squeeze the cilia and prevent them to beat efficiently. The purpose of the PCL is to act as a kind of lubricant which allows the mucus to slip onto it (Puchelle et al., 1995). Its thickness is around 6 μm. The mucus is composed of 95% of water, but also contains macromolecules called mucins (Lai et al., 2009). It is a highly non-Newtonian fluid which exhibits a plethora of complex properties such as visco-elacticity and thixotropy. Its role is to act as a barrier against the external environment and to trap the particles. Its depth varies between 5 and 100 μm depending on the position in the bronchial tree (Widdicombe and Widdicombe, 1995). One of the main difficulties met for its characterization is the huge variability of its rheological properties (Lafforgue et al., 2017). It can indeed vary by several orders of magnitude during the same day within a particular person (Kirkham et al., 2002).

In order to propel these two fluid layers, the epithelium is covered by tufts of cilia (around 200–300 cilia per tuft) which are cytoplasmic extensions put into motion by biochemical motors. Their motion can be decomposed into two steps: the stroke phase, which lasts around one third of the total beating period, where cilia will be almost orthogonal to the flow in order to maximize their pushing effect; and the recovery phase where cilia will bend themselves and get closer to the epithelial surface in order to minimize their impact on the flow. This spatial asymmetry is essential in the context of creeping flows, as it is the only mechanism that generates transport (Purcell, 1977; Khaderi et al., 2010). Note that the recovery phase does not occur in the same plane as the stroke phase, but instead occurs in a plane somehow more inclined in regards to the vertical axis (Sleigh et al., 1988). The cilia length is around 7 μm, thus allowing them to enter the mucus during the stroke phase. Cilia diameter is estimated to be around 0.2–0.3 μm according to Sleigh et al. (1988), and their beating frequency is around 15 Hz.

MCC can only work if both the mucus production and ciliary beating are fully functional. Indeed, diseases such as cystic fibrosis (CF), asthma, or Chronic Obstructive Pulmonary Disease (COPD), can all be related to abnormalities in the MCC process. In the case of CF, the mucus secreted is very viscous and in large quantities, which hinders the work of the cilia. Thus mucus flow becomes almost null and mucus accumulates. It leads to severe infections, which damage or destroy the cilia tufts. On the other hand, people with asthma have less cilia, and the ones remaining may be dysfunctional. The transport of mucus is obviously less efficient than for healthy persons, which is balanced by cough for instance.

Experimentally, it has been observed that cilia synchronize their beatings accordingly to their neighbors with a small phase lag (Sleigh, 1962). It results in metachronal waves (MCW) which can be seen at the surface formed by the cilia tips. When the phase lag ΔΦ between two cilia is negative, the MCW are called symplectic and move in the same direction as the flow. On the contrary, when 0 < ΔΦ < π, the MCW are called antiplectic and move in the direction opposite to the flow. These waves have been shown to greatly enhance the fluid transport (Gueron and Levit-Gurevich, 1999; Gauger et al., 2009), but there are still open questions on which kind of waves is the most efficient for mucus transport and mixing. Most of them are either experimental studies performed on living animals (Machemer, 1972), or numerical ones performed in a single fluid environment (Khaderi et al., 2011; Ding et al., 2014). Only few addressed the problem using a two-layer fluid (Chatelin and Poncet, 2016; Chateau et al., 2017). The main result of these works is that antiplectic MCW are found to be the most efficient, and that particular phase lags between two cilia maximize the mucus transport. Others authors (Sedaghat et al., 2016) have investigated the role of mucus rheology using a similar methodology as the one presented here, and found that the ratio of elastic contribution of mucus viscosity to the total mucus viscosity has a quite significant effect on the mucociliary transport. In particular, the mucus velocity was observed to increase when decreasing the elastic part of the mucus viscosity. The study of the mixing induced by beating cilia is also very important as it provides information about the deposition rate of particles (such as inhaled drugs) onto the epithelial cells. However, to the best of the author's knowledge, onlyDing et al. (2014) studied the mixing properties of both symplectic and antiplectic MCW but in a single fluid layer. The objective of the present paper is to fill this gap by having a deep insight into the transport and mixing properties of MCW in a more realistic two-phase environment.

The article is organized as follow: the algorithm used to model the MCC in a two-layer context is described in section 2. Results regarding the transport of passive tracers are presented in section 3, and a displacement ratio is introduced in order to quantify the efficiency of the wave organization. In section 4, the mixing capacities of the system are studied using tracers advection and by computing a global mixing index. Lyapunov exponents are also used in order to gain insight about how the mixing is locally achieved. Two time scales are also defined in order to compare the mixing induced by fluid advection to the mixing induced by molecular diffusion. Finally, conclusions summarize the main results of this work with some future views in section 5.

## 2. Numerical method

The Boltzmann equation describes the behavior of a gas from a microscopic point of view. The Lattice Boltzmann Method (LBM) solves the discrete Boltzmann equation for an ensemble of distribution functions *f*(*x, t*) on a discrete lattice. These distribution functions describe the probability that ensembles of particles, with velocity **e**_*i*_, collide and then stream along the discrete velocity vectors **e**_*i*_. By doing a Chapman-Enskog analysis, one can recover the Navier-Stokes equations as presented in Kruger et al. (2016) for instance. This kind of fluid solver is now considered as an efficient alternative to traditional Navier-Stokes solvers.

### 2.1. Mathematical description

#### 2.1.1. Single-component LB model

**Figure 1:**
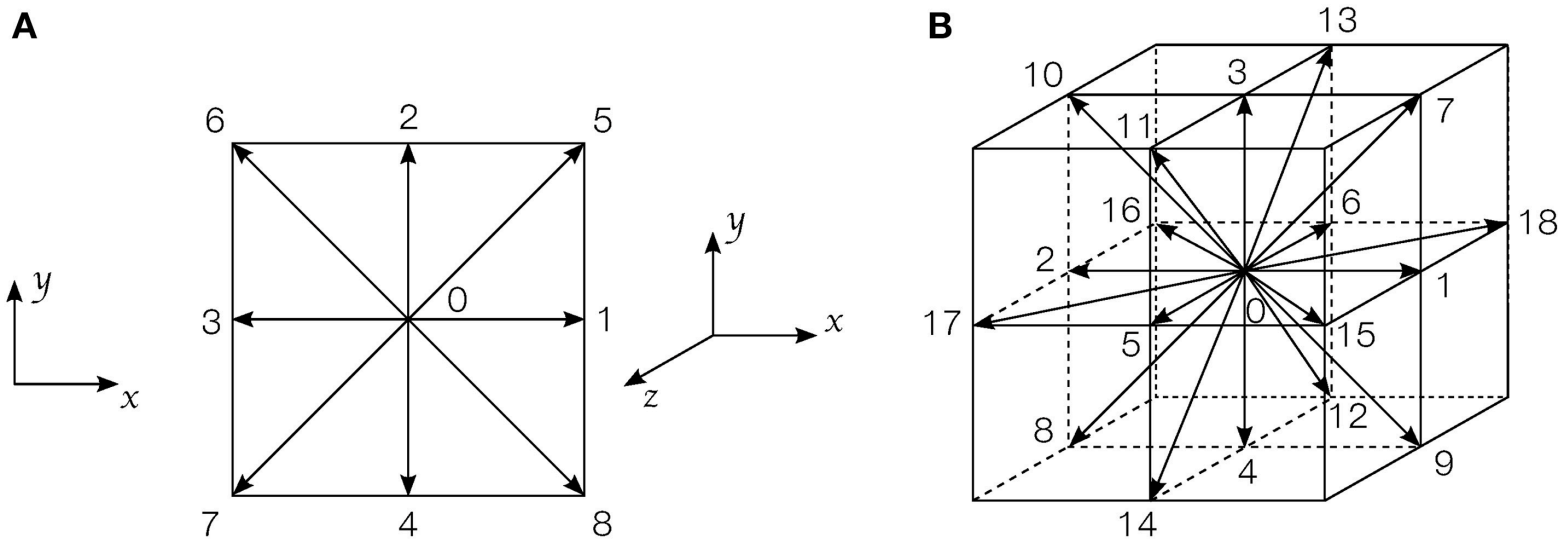
Discrete velocities arrangement on a lattice cell: **(A)** D2Q9 lattice; **(B)** D3Q19 lattice.

In LBM, the fluid status is updated in time by resolving the discrete Boltzmann equation (Chen and Doolen, 1998, and references therein):

(1) $$\begin{array}{r} f_{i}\left(\text{x}+{\text{e}}_{i}\Delta t,t+\Delta t\right)=f_{i}\left(\text{x},t\right)-\frac{\Delta t}{\tau }\left[f_{i}\left(\text{x},t\right)-f_{i}^{\left(eq\right)}\left(\text{x},t\right)\right] \end{array}$$

where *f*_*i*_(**x**, *t*) represents the distribution function at time *t* and position **x** in the *i*^*th*^ direction of the lattice (D2Q9 in 2D, and D3Q19 in 3D). Equation 1 uses the Single Relaxation Time (SRT) Bhatnagar-Gross-Krook (BGK) (Bhatnagar et al., 1954) collision operator. In this model, τ is the relaxation time, which is linked to the lattice viscosity by τ = 3ν+0.5 using the classical normalization procedure, i.e., Δ*x* = Δ*t* = 1 (Kruger et al., 2016). In this work, each phase is Newtonian, but has a different viscosity. The distribution functions move along a set of discrete velocity vectors **e**_*i*_, which depend on the lattice considered, as shown in Figure 1. The local density and momentum at each lattice node can be obtained by summing all the functions *f*_*i*_(**x**, *t*):

(2) $$\begin{array}{r} \rho \left(\text{x},t\right)=\overset{N}{\underset{i=0}{\sum }}f_{i}\left(\text{x},t\right)\;\rho \text{u}\left(\text{x},t\right)=\overset{N}{\underset{i=0}{\sum }}f_{i}\left(\text{x},t\right){\text{e}}_{i} \end{array}$$

where *N* is the number of discrete velocities on the lattice. The discrete equilibrium function $f_{i}^{\left(eq\right)}\left(\text{x},t\right)$, that appears in equation 1, can be obtained by Hermite series expansion of the Maxwell-Boltzmann equilibrium distribution (Chen and Doolen, 1998, and references therein):

(3) $$\begin{array}{r} f_{i}^{\left(eq\right)}=\rho \omega_{i}\left[1+\frac{{\text{e}}_{i}\cdot \text{u}}{c_{s}^{2}}+\frac{{\left({\text{e}}_{i}\cdot \text{u}\right)}^{2}}{c_{s}^{4}}-\frac{{\text{u}}^{2}}{c_{s}^{2}}\right] \end{array}$$

where $c_{s}=1/\sqrt{3}$ is the speed of sound in lattice unit. The weight coefficients ω_*i*_ are ω_0_ = 4/9, ω_1−4_ = 1/9 and ω_5−8_ = 1/36 for D2Q9 lattices, and ω_0_ = 1/3, ω_1−6_ = 1/18 and ω_7−18_ = 1/36 for D3Q19 lattices (Qian et al., 1992).

Body force effects are introduced by adding an extra term to Equation (1):

(4) $$\begin{array}{r} f_{i}\left(\text{x}+{\text{e}}_{i}\Delta t,t+\Delta t\right)=f_{i}\left(\text{x},t\right)-\frac{\Delta t}{\tau }\left[f_{i}\left(\text{x},t\right)-f_{i}^{\left(eq\right)}\left(\text{x},t\right)\right]+\Delta tF_{i}\left(\text{x},t\right) \end{array}$$

where *F*_*i*_ is given by the following equation:

(5) $$\begin{array}{r} F_{i}=(1-\frac{\Delta t}{2\tau })\;\omega_{i\;}\left[\frac{{\text{e}}_{i}-\text{u}}{2c_{s}^{2}}+\frac{{\text{e}}_{i}\cdot \text{u}}{c_{s}^{4}}{\text{e}}_{i}\right]\cdot \text{F} \end{array}$$

Here, **F** represents the body force per unit volume. The macroscopic velocity **u** must then be updated in order for the system to recover the Navier-Stokes equation:

(6) $$\begin{array}{r} \rho \text{u}=\underset{i}{\sum }{\text{e}}_{i}f_{i}+\frac{\Delta t}{2}\text{F} \end{array}$$

More details on the LBM model can be found in (Kruger et al., 2016, and references therein).

#### 2.1.2. Multi-component LB model

When considering two or more fluid components, the LB discrete equation is written as follows:

(7) $$\begin{array}{rcl} f_{i}^{\sigma }\left(\text{x}+{\text{e}}_{i}\Delta t,t+\Delta t\right) & = & f_{i}^{\sigma }\left(\text{x},t\right)-\frac{\Delta t}{\tau_{\sigma }}\left[f_{i}^{\sigma }\left(\text{x},t\right)-f_{i}^{\sigma \left(eq\right)}\left(\text{x},t\right)\right]\\ +\Delta tF_{i}^{\sigma }\left(\text{x},t\right) \end{array}$$

where $f_{i}^{\sigma }\left(\text{x},t\right)$ and τ_σ_ are the distribution functions and the single relaxation time of the σ^th^ component respectively. The expression of the equilibrium distribution function now reads:

(8) $$\begin{array}{r} f_{i}^{\sigma \left(eq\right)}=\rho_{\sigma }\omega_{i}\left[1+\frac{{\text{e}}_{i}\cdot {\text{u}}_{\sigma }^{\left(eq\right)}}{c_{s}^{2}}+\frac{{\left({\text{e}}_{i}\cdot {\text{u}}_{\sigma }^{\left(eq\right)}\right)}^{2}}{2c_{s}^{4}}-\frac{{\text{u}}_{\sigma }^{\left(eq\right)}\cdot {\text{u}}_{\sigma }^{\left(eq\right)}}{2c_{s}^{2}}\right] \end{array}$$

where $\rho_{\sigma }=\underset{i}{\sum }f_{i}^{\sigma }$ is the density of the σ^th^ component. ${\text{u}}_{\sigma }^{\left(eq\right)}$ is the equilibrium velocity which is identical for the two fluid components:

(9) $$\begin{array}{r} {\text{u}}_{\sigma }^{\left(eq\right)}={\text{u}}^{*}=\frac{\underset{\sigma }{\sum }\underset{i}{\sum }{\text{e}}_{i}f_{i}^{\sigma }/\tau_{\sigma }}{\underset{\sigma }{\sum }\underset{i}{\sum }f_{i}^{\sigma }/\tau_{\sigma }} \end{array}$$

In Equation (7), the explicit forcing term $F_{i}^{\sigma }$ is linked to the total body force **F**_σ_ per unit volume exerted on the σ^th^ component:

(10) $$\begin{array}{r} F_{i}^{\sigma }=\left(1-\frac{\Delta t}{\tau_{\sigma }}\right)\frac{{\text{F}}_{\sigma }\cdot \left({\text{e}}_{i}-{\text{u}}_{\sigma }^{\left(eq\right)}\right)}{\rho_{\sigma }c_{s}^{2}}f_{i}^{\sigma \left(eq\right)} \end{array}$$

Now, based on the methodology developed by Martys and Chen (2013), one adds a Shan-Chen-type fluid-fluid cohesion force ${\text{F}}_{\sigma }^{SC}$ in the total body force vector **F**_σ_ of Equation (10) in order to model the two-component behavior. The expression of the Shan-Chen type fluid-fluid cohesion force is (Shan and Chen, 1994):

(11) $$\begin{array}{r} {\text{F}}_{\sigma }^{SC}\left(\text{x},t\right)=-G_{coh}\rho_{\sigma }\left(\text{x},t\right)\underset{i}{\sum }\omega_{i}\rho_{\sigma^{\prime }}\left(\text{x}+{\text{e}}_{i}\Delta t,t\right){\text{e}}_{i} \end{array}$$

where *G*_*coh*_ is a parameter that controls the force of the cohesion force, and where σ′ represents a fluid different from σ. Note that with a Shan-Chen-type fluid-fluid cohesion force, there is no discontinuity of the fluid velocity at the interface, which is diffuse.

#### 2.1.3. The immersed boundary method

The aim of the IB method is to impose velocity boundary conditions on the Eulerian fluid nodes that surround a solid, by adding an extra body force ${\text{F}}_{\sigma }^{IB}$ to the fluid equations, so that the macroscopic fluid velocity can equal the velocity at the Lagrangian points modeling the solid boundary. Hence, an IB force ${\text{F}}_{\sigma }^{IB}$ is also included in the total body force vector **F**_σ_ so that ${\text{F}}_{\sigma }={\text{F}}_{\sigma }^{IB}+{\text{F}}_{\sigma }^{SC}$. The macroscopic velocity **u**_σ_ given by Porter et al. (2012) writes:

(12) $$\begin{array}{r} \rho_{\sigma }{\text{u}}_{\sigma }=\underset{i}{\sum }{\text{e}}_{i}f_{i}^{\sigma }+\frac{\Delta t}{2}{\text{F}}_{\sigma } \end{array}$$

The immersed boundary method to derive the forcing term uses the classical procedure which relies on two operators:

- The interpolation – In this step, the fluid velocity at the Eulerian nodes are used to perform an interpolation of the fluid velocity on the Lagrangian points.
- The spreading – An IB-related force is obtained as a function of the difference between the solid velocity and the interpolated fluid velocity. This force is spread to the surrounding Eulerian nodes in order to ensure the no-slip velocity condition at the fluid-solid boundary.

More details can be found in Li et al. (2016).

### 2.2. Modeling the MCC

**Figure 2:**
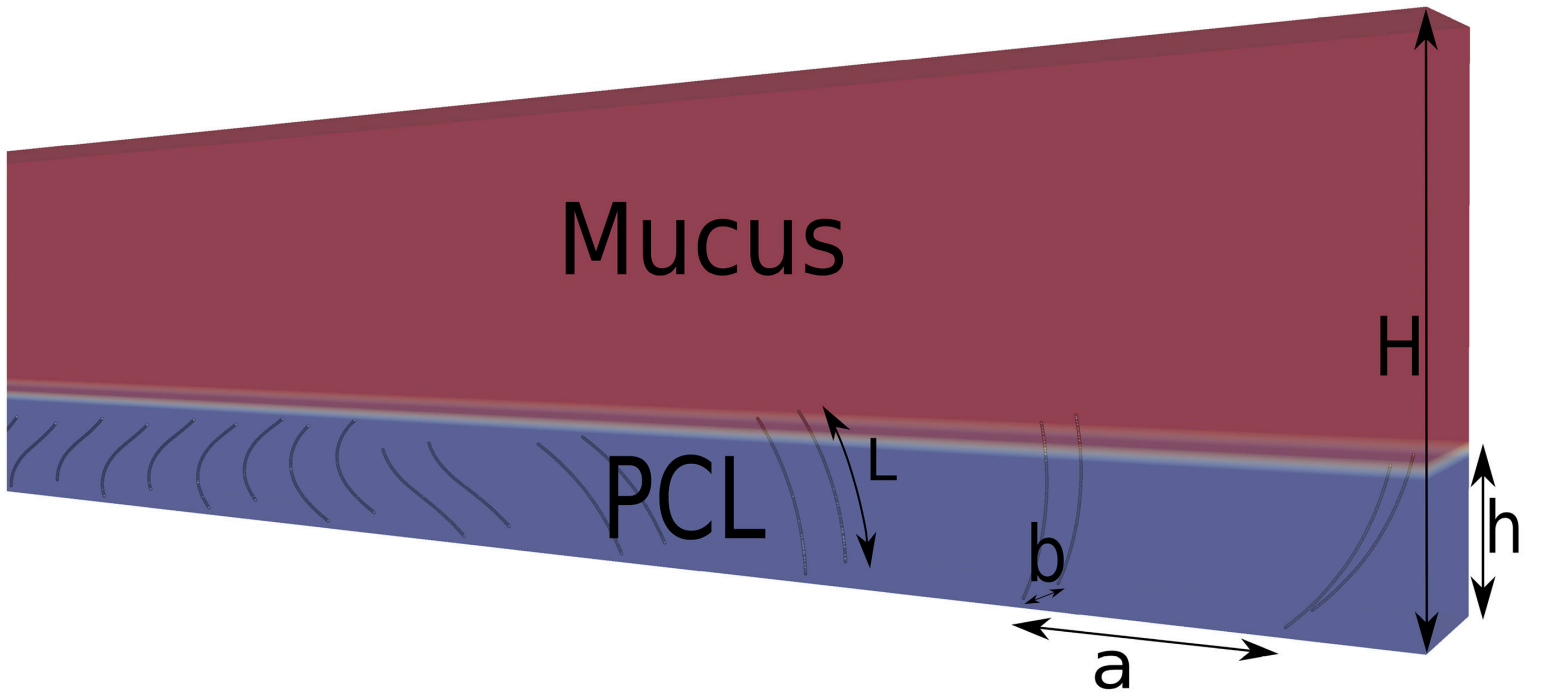
Schematic view of the domain. The length of the cilia is *L*, the cilia spacing in the *x*-direction is *a* = 1.44*L*, and in the *y*-direction is *b* = 0.4*L*. The ratio *h*/*H* is set to 0.26.

The computational domain is a fixed rectangular box of size (*N*_*x*_ = 385, *N*_*y*_ = 11, *N*_*z*_ = 34), as shown in Figure 2. The computational domain has been chosen as it allows to study the desired values of phase lags |ΔΦ| (ranging from ±π/6 up to ±π) without modifying the size of the domain and with a sufficiently-fine cilia resolution to ensure grid-independent results. The fluid part is solved on a Cartesian grid with a simple BGK collision operator, and a D3Q19 scheme. Periodic boundary conditions are used in the *x* and *y*-directions, while no-slip and free-slip boundary conditions are used at the bottom and top walls, respectively. The length *L* = 7 μm of the cilia is set to 11 lattice units (lu). Cilia are modeled by a set of 200 Lagrangian points, whose motion is governed by a differential 1D transport equation along a parametric curve (Chatelin, 2013; Chatelin and Poncet, 2016). In the following, *P*(ζ, *t*) denotes the position of the curve at time *t* and at a normalized distance ζ from the base point of a cilium. With appropriate boundary conditions, a realistic beating pattern is obtained:

(13) $$\frac{\partial P^{\prime }}{\partial t}+E(t)\frac{\partial P^{\prime }}{\partial \zeta }=0\text{ BC}:\{\begin{array}{l} P(0,t)=(0,0,0)\\ P^{\prime }(0,t)=(2\cos(2\pi t/T),0,\cos(2\pi t/T)) \end{array}$$

with *E*^2^(*t*) = ([1+8cos^2^(π(*t* + 0.25*T*)/*T*)]/*T*)^2^ a term which mimics elastic effects, *T* the beating period, and $P^{\prime }\;=\;\partial_{\zeta }P$. To ensure the stability of the IB method, there must be approximatively one Lagrangian point per lattice cell where the IB forces are computed. Thus only 10 Lagrangian points regularly spaced onto the cilia are chosen for the computation of the IB forces. The spacing between two cilia is set to *a* = 1.44*L* in the *x*-direction, and *b* = 0.4*L* in the *y*-direction. Their base point is located at *z* = 0 which corresponds to the position of the epithelial surface. The beating period is *T*_*osc*_ = *N*_*it*_Δ*t*, where *N*_*it*_ is the number of iterations for performing a full beating cycle. The PCL fills the domain from *z* = 0 up to an altitude *z* = *h* = 0.9*L*. In all simulations, *N*_*z*_ is fixed to 34 lu, leading to a ratio *h*/*H* = 0.26. The wavelength of the imposed metachronal waves varies from λ = 32 lu for a phase lag ΔΦ = π, to λ = 192 lu for ΔΦ = π/6.

The motion of the cilia is imposed to be in the *x*-direction only. Note that, due to the inter-cilia spacing, no collision between cilia occurs during their beatings. Since the only mechanism to impose motion in creeping flow is the spatial asymmetry (Purcell, 1977; Khaderi et al., 2010), no temporal asymmetry is considered in the beating pattern. The viscosity of the PCL is chosen to be $\nu_{PCL}=10^{-3}$ m^2^/s, and the ratio of viscosity *r*_ν_ between the mucus and PCL is set to 10. Since the model of Porter et al. (2012) introduces a Shan-Chen fluid-fluid repulsive force (Shan and Chen, 1994), surface tension effects emerge intrinsically at the mucus-PCL interface. More importantly, this also prevent the mixing of the mucus and PCL. The equations of the cilia motion are taken from Chatelin (2013) and reproduce a 2D beating pattern similar to the one observed for real cilia. In particular, the angular amplitude of this beating pattern is θ = 2π/3 as observed experimentally (Sleigh et al., 1988). Thus, the velocity *U*_*cil*_ at the tips of the cilia can be computed by *U*_*cil*_ = 2θ*L*/*T*_*osc*_, and an oscillatory Reynolds number can be defined as:

(14) $$\begin{array}{r} Re^{osc}=\frac{U_{cil}L}{\nu_{mucus}}=\frac{\omega L^{2}}{\nu_{mucus}} \end{array}$$

where ω is the angular beating frequency of cilia. Using physical quantities ($L_{phy}\approx 10^{-5}$ m, $\nu_{mucus}\approx 10^{-3}$ m^2^.s^−1^, and $U_{cil}\approx 10^{-3}$ m.s^−1^), the obtained Reynolds number is of the order of 10^−5^. Thus, inertial effects do not play any role in the phenomenon of MCC. Running simulations at such a low Reynolds number would require a huge number of iterations using a lattice Boltzmann scheme due to the coupling between Δ*x* and Δ*t* imposed by the normalization. Hence, we chose higher Reynolds numbers: *Re*^*osc*^ = 2.10^−2^, 5.10^−2^, and 10^−1^, as it has been demonstrated in Chateau et al. (2017) that inertial effects remain weak in this configuration up to Reynolds numbers around 10. For Re = 10^−2^, inertia effects vanish. In creeping flow, there should be no noticeable difference in the wave structure even for a Reynolds number 1,000 times weaker. The code is parallelized using MPI (Message Passing Interface) by splitting the computational domain into 9 subdomains of size (*N*_*x*_/3, *N*_*y*_/3, *N*_*z*_).

## 3. Mucus transport

**Figure 3:**
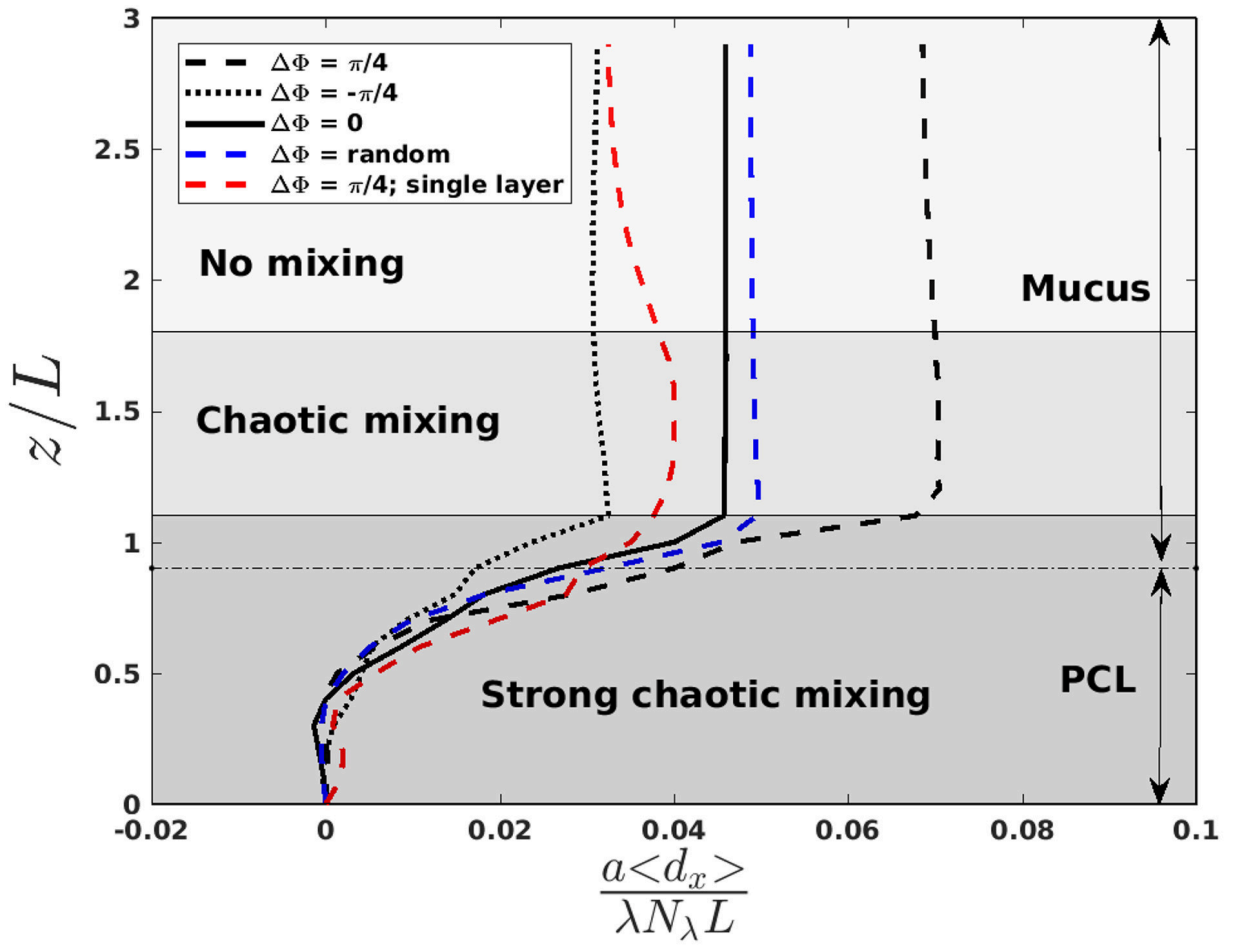
Normalized averaged displacement of fluid in the *x*-direction as a function of *z*/*L* with *a*/*L* = 1.44 and *b*/*L* = 0.4, for a Reynolds number *Re* = 10^−1^. λ is the wavelength of the MCW, and *N*_λ_ the number of cilia in one wavelength. The “strong chaotic mixing” zone extends from 0 to 1.1*L*, the “chaotic mixing” zone from 1.1 to 1.8*L*, and the “no mixing” zone from 1.8 to 3*L*. The mucus-PCL interface is located around *z*/*L* = 0.9 and indicated by a dashed horizontal line. The red curve corresponds to a simulation performed in a single layer fluid with a viscosity ν = ν_*mucus*_. The blue curve corresponds to a simulation where each cilium has been initially set in a random state of beating: the phase of the cilia being uncorrelated. The black curves correspond to antipleptic MCW (ΔΦ = π/4), symplectic MCW (ΔΦ = −π/4), and synchronous beatings (ΔΦ = 0). More details regarding how the chaotic zones were defined are given in section 4.

A common way to treat respiratory diseases is by the inhalation of drugs, which flow into the airways until they are captured by the mucus layer. To gain an insight into how drugs are dispersed and advected into the mucus and PCL, the displacement field $d(x)=\int_{0}^{{\text{T}}_{osc}}u(x(t),t)dt$ is computed, where **x** is the position vector and **u** is the fluid velocity. The component over the *x*-direction of the displacement field is then averaged over 20 beating periods and denoted < *d*_*x*_ >. It is plotted on Figure 3. One can clearly see the importance of the phase lag, some values being associated to larger displacement of fluid. One can also observe that the particular case where all the cilia beat synchronously (i.e., ΔΦ = 0) results in a transport which is similar to the action of fully desynchronized cilia (i.e., ΔΦ between two neighboring cilia is random). Note that to test the repeatability of the random motion, three simulations with an initially different random pattern were performed. Each of them gave almost identical results, with less than 3% of difference in the fluid velocity. In order to understand why the presence of the PCL layer is beneficial for the mucus transport, a simulation of a single-fluid layer, representing mucus, has been run for a phase lag ΔΦ = π/4 (see the red curve in Figure 3). It results in a weaker transport compared to the corresponding two-layer fluid simulation with ΔΦ = π/4, thus highlighting the importance of having a layer of fluid with lower viscosity under the mucus one as it allows the mucus to slip onto it (Puchelle et al., 1995). In Figure 3, different areas, corresponding to different mixing regimes, are also presented and will be introduced later in section 4. These regions are similar to the “transport” and “mixing” areas defined in Ding et al. (2014) and Chateau et al. (2017). The displacement over the *y* and *z*-directions has also been quantified. The displacement in the *y*-direction is small everywhere, and thus can be neglected. On the contrary, the displacement in the *z*-direction is small above the cilia tips, but not under. It has been shown in Chateau et al. (2017) that a peak in the stretching rate is present in this region. It will be shown in section 4 that it is also the area where the mixing is the strongest.

**Figure 4:**
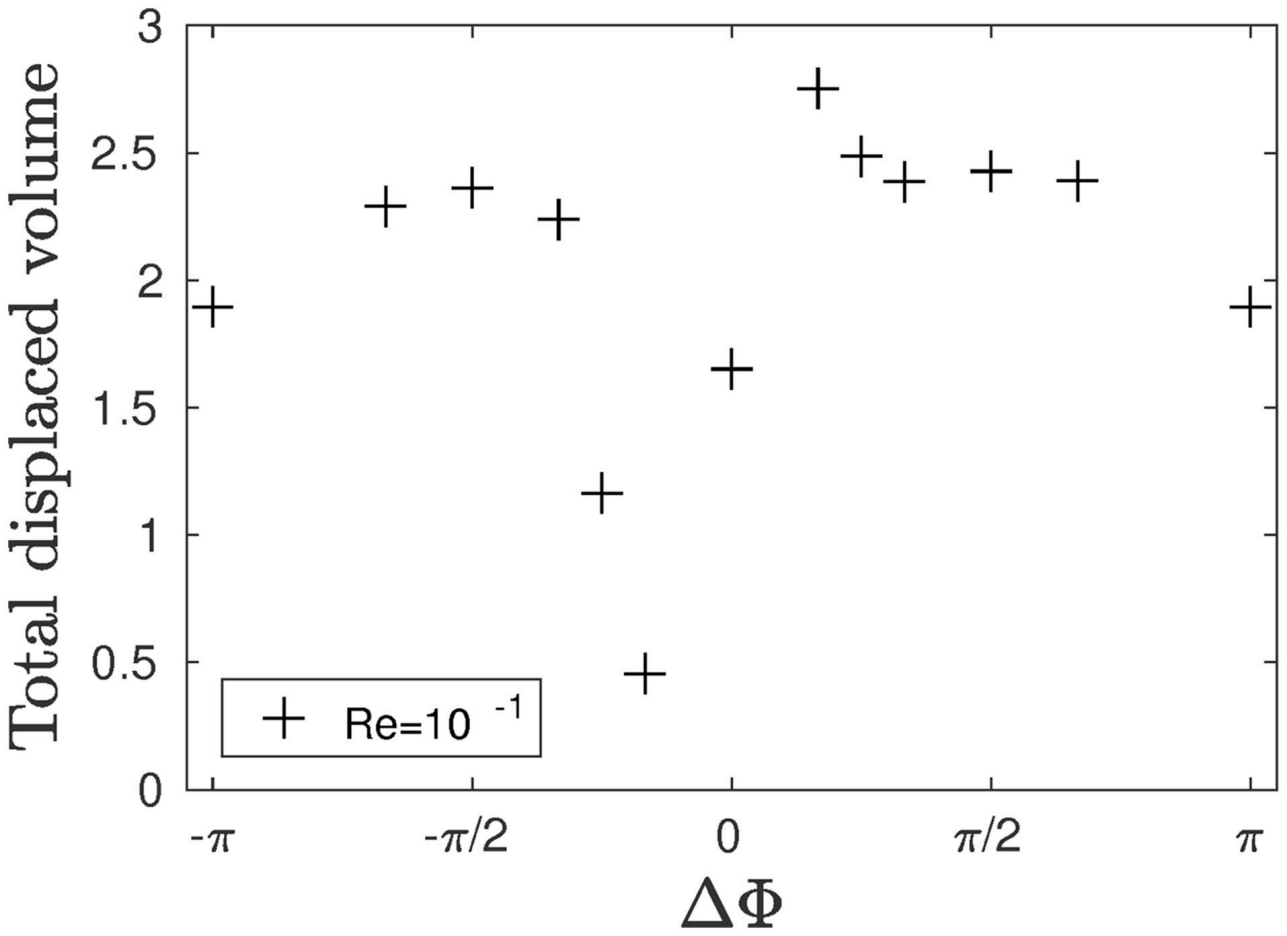
Total dimensionless displaced flow volume generated by an array of 24 × 2 cilia over a beating cycle for different phase lags ΔΦ and Re = 10^−1^.

The total volume of fluid effectively displaced is computed in order to determine which phase lag is more able to transport the mucus. To do so, the global volumetric flow rate *Q*_*v*_ over a unit volume of size (1 × 1 × *N*_*z*_) is defined by:

(15) $$\begin{array}{r} Q_{v}=N_{z}\frac{U^{*}\Delta x^{2}}{L^{2}} \end{array}$$

with U^*^ = *U*^*av*^/*U*^*ref*^, where $U^{ref}=\lambda /\left(N_{cil}T\right)$ is the reference velocity of the system, and $U^{av}={\left(N_{x}N_{y}N_{z}\right)}^{-1}\underset{i,j,k}{\sum }U_{ijk}$ is the average fluid velocity inside the domain. The result for the total displaced volume of fluid is plotted in Figure 4. Metachronal motion, except for the cases where ΔΦ = −π/6 and ΔΦ = −π/4, induces a stronger displacement of fluid compared to the synchronized motion (ΔΦ = 0). Note that the results for ΔΦ = −π/6 and ΔΦ = −π/4 slightly differ from what is found in Chateau et al. (2017) where, for Reynolds numbers of the order of 10^−2^, symplectic MCW were found to be more efficient than synchronized motion. This is a direct consequence of the modified geometry: indeed, in Chateau et al. (2017), the cilia spacing *b* in the *y*-direction was set to values larger than 1.67*L*. Thus, during the stroke phase of symplectic MCW (which corresponds to a moment where the cilia are being clusterized), the fluid flow was simply expelled around the cilia. In the present case, *b* is much smaller (*b* = 0.4*L*) in order to have a higher density of cilia as observed in real epitheliums, and the fluid is mainly pushed above the cilia. It results in a displacement of the mucus-PCL interface above the cilia tips which never get the chance to enter the mucus layer. On the contrary, the cilia during the recovery phase are far away from each other. A suction effect occurs, leading the mucus-PCL interface to be moved downwards toward the cilia. Thus, the counter flow created by the cilia during the recovery phase is almost as strong as the flow created by the cilia during the stroke phase. As a consequence, both the PCL and mucus flows are much smaller. The opposite happens for antiplectic MCW with large wavelengths (i.e., small ΔΦ): the cilia are far from each other during the stroke phase, which maximizes their pushing effect. The suction effect also takes place, which results in the mucus-PCL interface moving downwards. Hence, the cilia tips penetrate more deeply into the mucus phase. During the recovery phase, the cilia are now clusterized, and the mucus-PCL interface is pushed far away from the cilia tips. Hence, the induced counter flow is almost null, while the cilia during the stroke phase creates a strong positive flow. This result is interesting as it might be linked to the fact that antiplectic MCW with very large wavelength (ΔΦ < π/6) are usually observed in nature for living organisms evolving in single layer fluid environments (Sleigh, 1962). This blowing and suction mechanism is similar to the one observed in Dauptain et al. (2008) on a similar configuration involving the swimming of a jellyfish by ciliary propulsion. A maximum in the total fluid displaced volume can be seen in Figure 4, and corresponds to an antiplectic MCW with ΔΦ ≈ π/6, which corroborates the results found in Chateau et al. (2017) for antiplectic MCW where a peak in the total displaced fluid volume was found for ΔΦ ≈ π/4.

**Figure 5:**
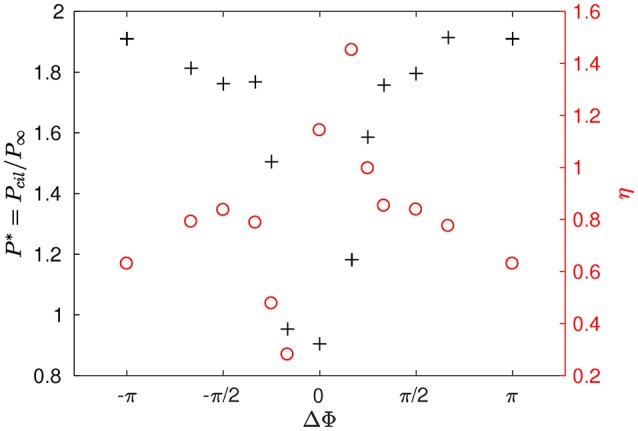
Power P^*^ spent by the system and displacement ratio η obtained for different phase lags ΔΦ and Re = 10^−1^.

In order to characterize the system from an energy perspective, the average power *P*_*cil*_ spent by the cilia during a beating cycle is introduced:

(16) $$\begin{array}{r} P_{cil}=\frac{\underset{s,i}{\sum }{\text{V}}_{i}^{s}\cdot \left({\text{F}}_{m}^{i}+{\text{F}}_{PCL}^{i}\right)}{N_{cil}} \end{array}$$

where ${\text{V}}_{i}^{s}$ is the velocity on the *s*th Lagrangian points of the *i*th cilium, and ${\text{F}}_{m}^{i}$ and ${\text{F}}_{PCL}^{i}$ the interpolated IB forces, respectively applied by the *i*th cilium onto the mucus and PCL. In order to have a dimensionless power P^*^, *P*_*cil*_ is normalized by *P*^∞^ the power spent by an isolated cilium during a beating cycle (*a*/*L* = *b*/*L* = 5), such that $P^{*}=P_{cil}/P^{\infty }$. The displacement ratio η can now be defined as the mean displacement over the *x*-direction divided by the mean power a cilium has to spend during a beating cycle:

(17) $$\begin{array}{r} \eta =\frac{<d_{x}^{*}>\frac{N_{cil}}{\lambda }}{P^{*}} \end{array}$$

where $<d_{x}^{*}>$ is the mean displacement over the *x*-direction during one period, taken on an arbitrary plane (*z*/*L* = 3.2) near the top of the domain. The left axis of Figure 5 shows the dimensionless power P^*^ spent by the system. The synchronized case requires less energy than other type of coordinated motion. Note that MCW with a phase lag such that π/3 < |ΔΦ| < π result in the highest power spent, while smaller phase lags (|ΔΦ| < π/4) require less energy. On the right axis of Figure 5, one can observe the variations of the displacement ratio η. For a given power input, the synchronized motion of the cilia is almost always more efficient than MCW for displacing fluids, except for antiplectic MCW with ΔΦ = π/6. This result can explain why antiplectic MCW with large wavelengths are usually observed in nature.

## 4. Mixing

### 4.1. Global mixing

**Figure 6:**
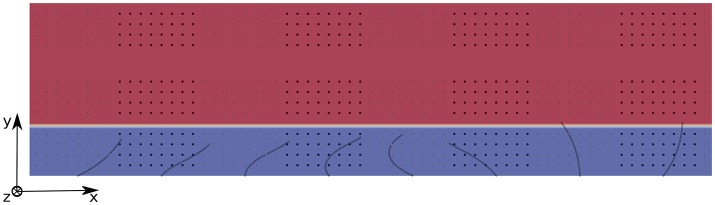
2D view of the domain filled with 3 populations of tracers for Re = 5.10^−2^. The PCL is blue, and the mucus phase is red. Population 1 occupies the PCL between *z* = 0.2*L* and *z* = 0.8*L*; Population 2 is located above the PCL-mucus interface and occupies the region between *z* = 1.2*L* and *z* = 1.8*L*; Population 3 is located far above the PCL-mucus interface, and occupies the region between *z* = 2.5*L* and *z* = 3.1*L*. The size of the computational domain is (*N*_*x*_ = 385, *N*_*y*_ = 11, *N*_*z*_ = 34).

**Figure 7:**
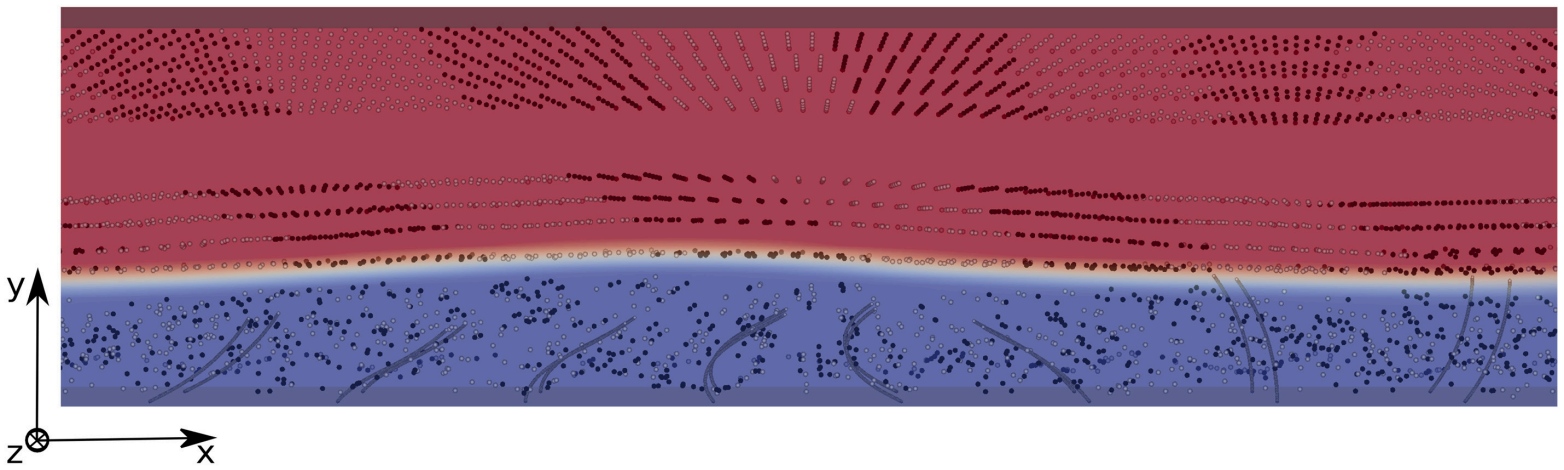
3D view of the domain filled with 3 populations of tracers for Re = 5.10^−2^, 60 beating cycles after their release at *t* = *t*_0_ when the flow is fully-established. The tracers in the PCL are significantly mixed, while the tracers in areas 2 and 3 still present coherent patterns.

The mixing is quantified using the method developed in Stone and Stone (2005): two populations of tracers of different colors (black and white) are initially organized in a regular pattern; each population occupying the same volume (see Figure 6 for a view of the domain filled with tracers). They are released at *t* = *t*_0_ when the flow is fully-established, and a second order Runge-Kutta (RK2) scheme is used to compute their advection, using the interpolated fluid velocity given by the IB method. The mixing is quantified by measuring the decay of the shortest distance between tracers that belong to the different populations. Hence, the mixing number *m* is defined as follows:

(18) $$\begin{array}{r} m={\left(\overset{N}{\underset{i=1}{\prod }}\text{min}{\left(\left|{\text{x}}_{i}-{\text{x}}_{j}\right|\right)}^{2}\right)}^{\frac{1}{N}} \end{array}$$

where **x**_*i*_ and **x**_*j*_ are the positions of tracers of different colors, *N* is the total number of particles of the same color, and *j* = 1, 2, …, *N* is the index for which the minimization is performed. We chose to study the mixing in three different areas: area 1 is located inside the PCL, and the tracers are set such that they occupy the region between *z* = 0.2*L* and *z* = 0.8*L*; area 2 is located above the PCL-mucus interface and the tracers occupy the region between *z* = 1.2*L* and *z* = 1.8*L*; and area 3 is located far above the PCL-mucus interface, and the tracers occupy the region between *z* = 2.5*L* and *z* = 3.1*L* (see Figure 6 for a view of the different areas). The chosen pattern consists in rectangular boxes of size (1.44*L*, 0.4*L*, 0.6*L*) regularly distributed along the *x*-direction, each of them being centered around the base of a cilium. This geometrical distribution has been chosen in order to provide comparative results with Ding et al. (2014). The density of tracers is not a critical factor here, as pointed out by Stone and Stone (2005). Hence, in each area one tracer is placed every 2 nodes along the three directions of space. On Figure 7, the different mixing areas are displayed after 60 beating cycles for a Reynolds number of Re = 5.10^−2^: the tracers initially seeded into the PCL are significantly mixed, contrarily to the tracers initially seeded far above the mucus-PCL interface. Between these two populations, the tracers initially seeded just above the mucus-PCL interface undergo a constant shearing. It is worth noticing that tracers initially seeded into the PCL (resp. mucus) stay in the PCL (resp. mucus). This behavior is attributed to surface tension effects present at the interface which prevent a mixing of the two fluid layers. This shows that particles captured by the mucus layer will never reach the PCL. However, note that the present model does not take into account molecular diffusion effects, which may allow drugs to penetrate the PCL area. Nevertheless, the effects of diffusion will be considered in section 4.3 using two different time scales.

**Figure 8:**
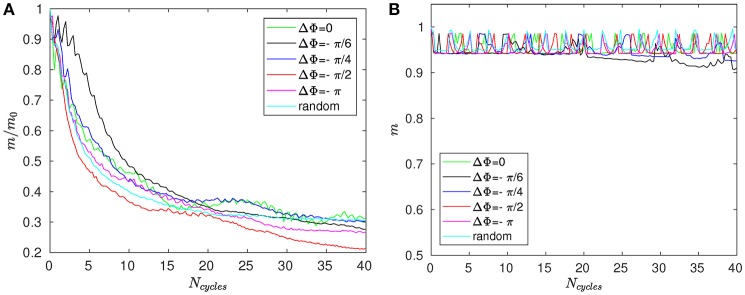
Mixing number *m*/*m*_0_ as a function of the number of cycles *N*_cycles_ for Re = 2.10^−2^ and different phase lags ΔΦ in **(A)** area 1 (i.e., within the PCL) and **(B)** in area 3 (far above the mucus–PCL interface).

**Figure 9:**
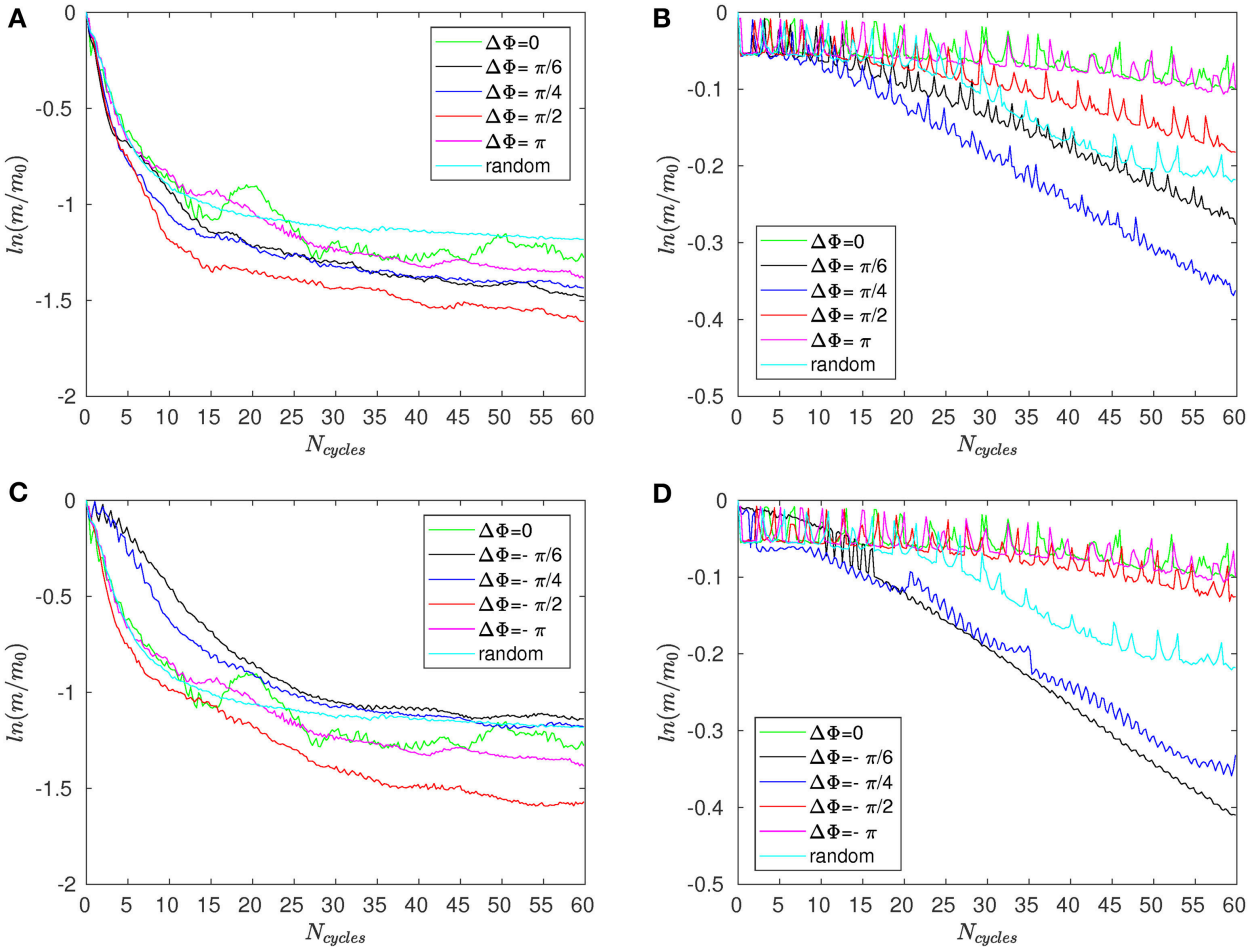
Logarithm of the mixing number, ln(*m*/*m*_0_), as a function of the number of cycles *N*_cycles_ for Re = 5.10^−2^ and for different phase lags ΔΦ corresponding to antiplectic MCW in **(A)** the PCL region (area 1); and **(B)** above the mucus–PCL interface (area 2). **(C,D)** are similar to **(A,B)**, but for symplectic MCW.

**Figure 10:**
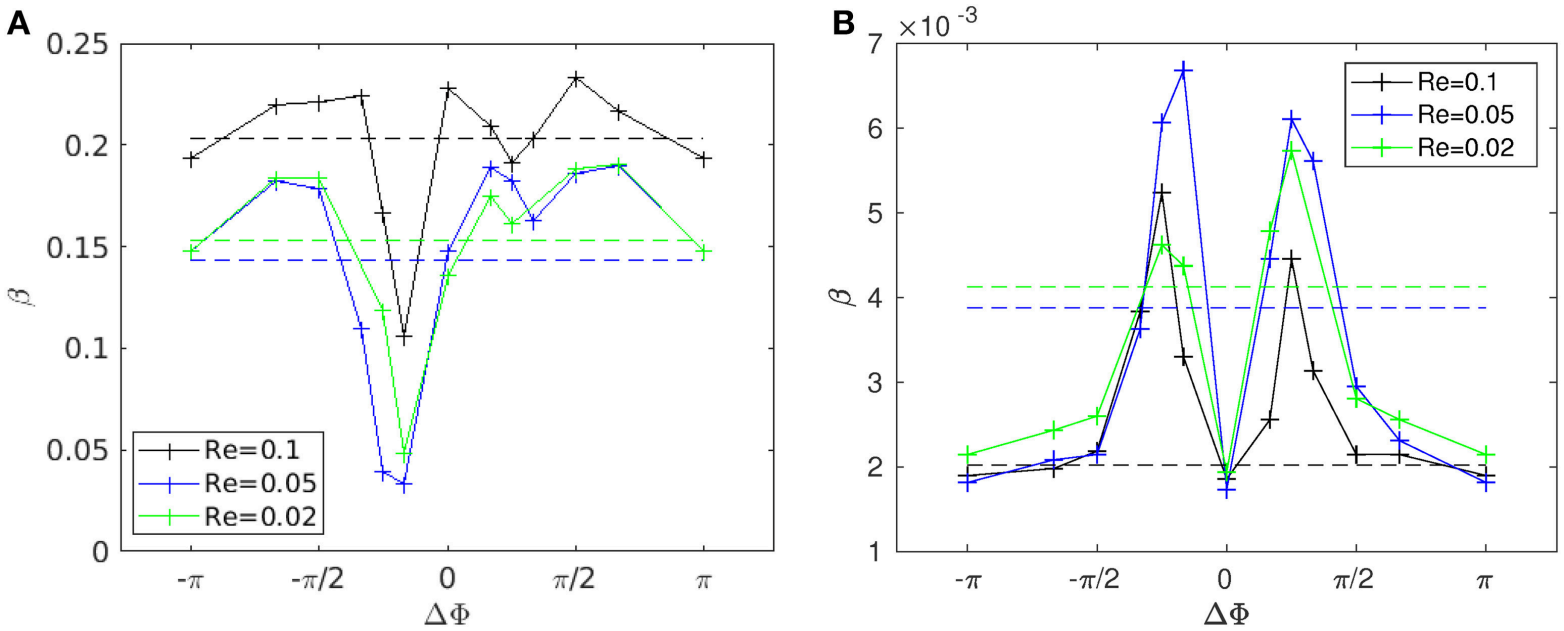
Mixing rate β for different Reynolds numbers (Re = 2.10^−2^, 5.10^−2^, and 10^−1^) as a function of the phase lag ΔΦ. **(A)** Mixing rate obtained in area 1 with a fit over the 4 first beating cycles. **(B)** Mixing rate obtained in area 2 with a fit over all 60 beating cycles. The dashed lines represent the values of the mixing rate obtained for cilia beating randomly. Note that the *y*-scales used in **(A,B)** are different: the values of β corresponding to area 1 are of order 10^2^ times greater than those obtained in area 2. Also note that the repeatability of the random motion has been tested, and similar values for β with less than 2% of difference were found.

Figure 8A shows the time evolution of the mixing number *m*/*m*_0_ in the PCL (area 1) for different metachrony; *m*_0_ denoting the initial value of *m* when the tracers are not yet released. If the mixing is chaotic, the mixing number *m*/*m*_0_ should be decaying exponentially. It is indeed the case during the first beating cycles (5–6 cycles). However, if only chaotic mixing was present, the measures would simply converge toward a “plateau.” This is not the case here as the cilia also impose a stretching to the generated flow. Thus, the ratio *m*/*m*_0_ keeps on decaying and converge only toward a “pseudo-plateau.” Since we are mainly interested by a characterization of the chaotic mixing induced by cilia, we will focus our attention to the first beating cycles. Figure 8B confirms that the mixing in the mucus is very low. The mixing number *m*/*m*_0_ is almost constant during all 60 beating cycles. The tracers are transported as a solid block and keep their initial pattern, as illustrated in Figure 7. On Figure 9A, the logarithm of the dimensionless mixing number *m*/*m*_0_ in area 1 is plotted. The fact that *m* decays rapidly means that the mixing in this area is strong: indeed, only 4 beating cycles are required to obtain a converged state of mixing. During these first beating cycles, the decay of *m* strongly depends on the value of the phase lag ΔΦ. The results for symplectic MCW (ΔΦ < 0) are similar to those obtained for antipleptic MCW (see Figure 9A,C). On Figure 9B,D, the same quantities are plotted for area 2. One can observe the importance of ΔΦ, some phase lags being clearly more able to mix the tracers. It is interesting to note that each curve presented in Figure 9A,B exhibits the behavior of chaotic mixing. In other words, they can be approximated by a function of form ln(*m*/*m*_0_) = −β*N*_*cycles*_, where the fitted parameter β represents a mixing rate, which depends on the local stretching rate (Weiss and Provenzale, 2007). Hence, it is possible to compare the mixing capabilities of symplectic and antiplectic MCW, as shown in Figure 10A,B. The mixing rates β obtained for three different Reynolds number (Re = 10^−1^, Re = 5.10^−2^, and Re = 2.10^−2^) are plotted as a function of ΔΦ for areas 1 and 2 respectively (see Figure 10A). The curves follow the same trend for each value of tested Reynolds number. There are always values of ΔΦ ≠ 0 such that the obtained mixing rate β is superior to the synchronized case; except for the case Re = 0.1 where the value of ΔΦ = 0 induces a mixing rate β almost as strong as for ΔΦ = π/2. As seen in section 3, the values of ΔΦ = −π/6, and ΔΦ = −π/4 induce a weak mixing. This is the direct consequence of the fact that the PCL-mucus interface is pushed above the cilia tips during their stroke phase, which hinders them to penetrate the mucus layer. As a result, the fluid flow is weaker in both the PCL region and mucus region. In Figure 10B two distinct peaks can be identified, one for antiplectic MCW with ΔΦ ≈ π/4, and the second for symplectic MCW with ΔΦ ≈ −π/4, indicating that these particular values of phase lag are more efficient to mix the mucus. While the value of ΔΦ ≈ −π/4 induces a small transport of the PCL and mucus, and a small mixing of the PCL, it is interesting to note that it can generate a mixing as strong as the case ΔΦ ≈ π/4 above the PCL-mucus interface. In both cases (ΔΦ = ±π/4), this can be attributed to the motion of the interface. Note that too large values of ΔΦ induce a mixing which is similar to the one of synchronized beating cilia (ΔΦ = 0). Also, the *y*-scale of Figure 10B is much smaller (100 times smaller) than the one of Figure 10A. In both Figure 10A,B, the dashed lines represent the mixing rates β obtained for cilia beating randomly. The mixing rate obtained for such configuration may vary depending on the initial conditions of the cilia, but not significantly, as it is “averaged” over the random motion of 48 cilia. Interestingly, the motion of randomly beating cilia produces an “averaged” mixing rate: although never being in the highest values of β, it always induces a mixing reasonably high.

The conclusion here is that the mixing induced by MCW in area 1 is very similar to the mixing induced by synchronized motion, except for the particular case of symplectic MCW with very large wavelengths. In area 2, specific values of phase lags are found to be more efficient to mix the mucus-PCL area compared to synchronized or random beatings. Finally, in area 3, the mixing is weak and independent of the phase lag ΔΦ.

### 4.2. Local mixing

Specific drugs, such as the propranolol (PPL) or β-adrenergic, act on the cilia by modifying their beating frequency (Inoue et al., 2013). Others, such as the anticholinergics or the corticosteroids, act directly on the mucus secretion (Barnes, 2002). Each of these drugs have specific targets, and must arrive precisely where they will have the most effects. Hence, it is important to fully understand how they will be mixed. However, many questions remain open: Where are the drugs mainly mixed ? Where exactly is the location of strongest mixing in the PCL ? To answer these questions, a different method is now introduced in order to measure locally the mixing, and gain a detailed insight into how the particles are mixed depending on their location. To do so, the methodology used in Cieplak et al. (1992) is adopted. The principle is simple: one must follow the evolution of the distance *r* between tracers initially separated by an infinitesimal distance *r*_0_. In the particular case of chaotic mixing, a Lyapunov exponent γ can be extracted using the following equation:

(19) $$\begin{array}{r} \ln\left(\frac{r}{r_{0}}\right)=\gamma N_{cycles} \end{array}$$

This exponent gives an indication on the strength of the mixing. However, a sufficiently high number of measurements must be performed to get rid of the noise inherent to this method. To do so, a cubic set of (3 × 3 × 3) tracers, referred later as “fathers,” are used. These fathers are initially set at a distance *r*_0_ = 0.01 lu apart from each other. For each father, 6 tracers, referred from now as “children,” are regularly initialized around the fathers along the 3 directions of space at a distance of 0.001 lu. Thus, 162 pairs of tracers are considered and their average distance *r*_mean_ is regularly computed during several beating cycles.

Five typical positions are studied:

- Position A, located at (*a*/2, *N*_*y*_/4, 0.45*L*), where the tracers are in the middle of the PCL, and onto the trajectory of a cilium.
- Position B, located at (*a*/2, *N*_*y*_/4, *L*), where the tracers are just above the PCL-mucus interface, and above a cilia.
- Position C, located at (*a*/2, *N*_*y*_/4, 2*L*), where the tracers are far into the mucus layer (1*L* above the tracers of position B).
- Position D, located at (*a*/2, *N*_*y*_/2, 0.45*L*), where the tracers are in the middle of the PCL between two cilia along the *y*-direction.
- Position E, located at (*a*/2, *N*_*y*_/4, 0.1*L*), where the tracers are just above the epithelial surface, and onto the trajectory of a cilium.

**Figure 11:**
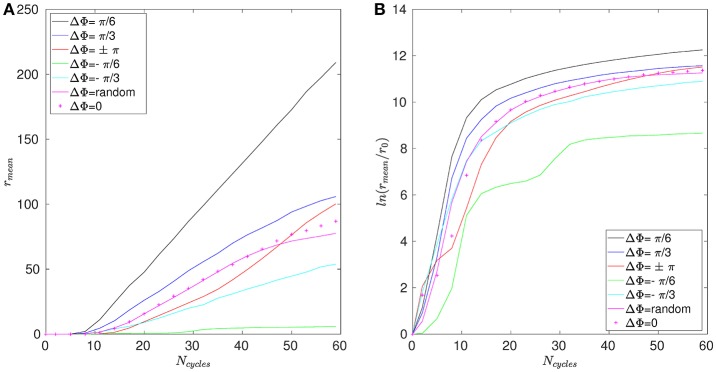
Results obtained for position A and Re = 5.10^−2^. **(A)** Average distance *r*_*mean*_ between the fathers and the children as a function of the number of cycles *N*_*cycles*_. **(B)** Logarithm of the dimensionless average distance *r*_*mean*_/*r*_0_ as a function of the number of cycles *N*_*cycles*_.

**Figure 12:**
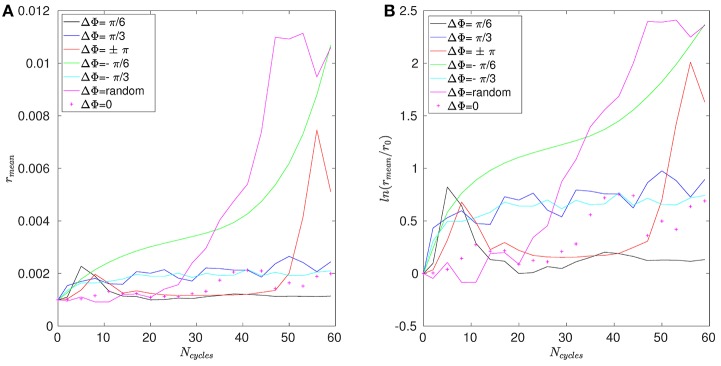
Results obtained for position B and Re = 5.10^−2^. **(A)** Average distance *r*_*mean*_ between the fathers and the children as a function of the number of cycles *N*_*cycles*_. **(B)** Logarithm of the dimensionless average distance *r*_*mean*_/*r*_0_ as a function of the number of cycles *N*_*cycles*_.

**Figure 13:**
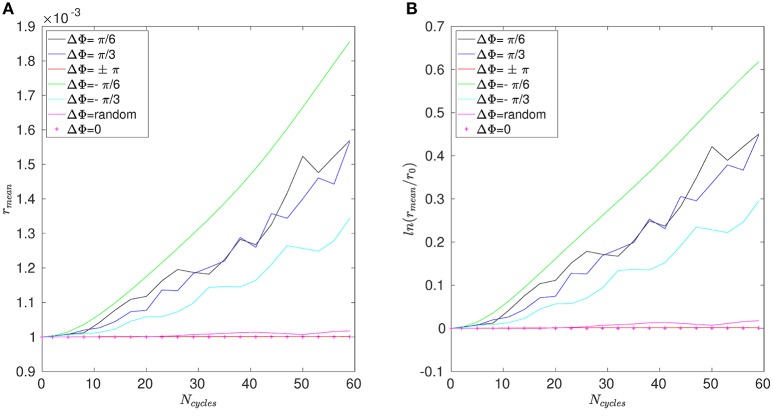
Results obtained for position C and Re = 5.10^−2^. **(A)** Average distance *r*_*mean*_ between the fathers and the children as a function of the number of cycles *N*_*cycles*_. **(B)** Logarithm of the dimensionless average distance *r*_*mean*_/*r*_0_ as a function of the number of cycles *N*_*cycles*_.

**Figure 14:**
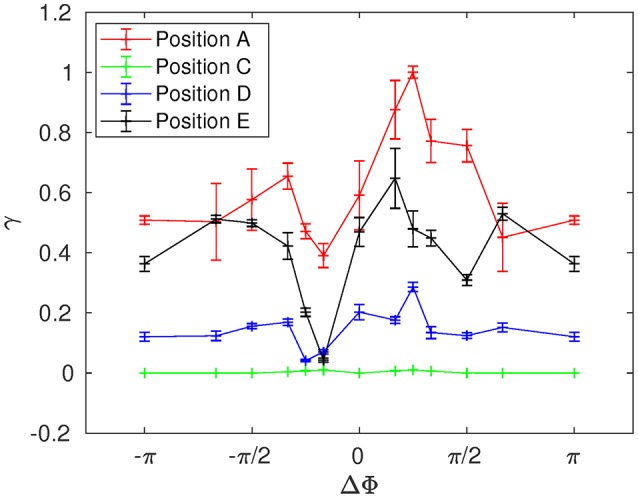
Lyapunov exponent γ as a function of the phase lag ΔΦ for Re = 5.10^−2^ at positions A, C, D, and E.

The mean distance *r* for positions A, B, and C are given in Figures 11–13. Note that the results for positions D and E are not displayed in the following, as they are very similar to those obtained for position A. In Figure 11A, one can see the evolution of the average distance *r*_*mean*_ as a function of the number of cycles *N*_*cycles*_ for several phase lags ΔΦ. It takes around 10 cycles for the distance between fathers and children to significantly increase. One can see in Figure 11B that the evolution of ln(*r*_*mean*_/*r*_0_) is linear during the first cycles, indicating chaotic mixing. Similar results are obtained for positions C, D, and E (see Figure 13 for position C). Thus, we can extract Lyapunov exponents for each curve by considering only their linear parts. It is important to note that, while the measures indicate chaotic mixing only during the first beat cycles after the tracers release, the mixing is *always* chaotic: indeed, the flow is well-established and its properties do not change over time. For position B (see Figure 12A,B), the tracers are initialized at 1*L* (thus 0.1*L* above the interface), and no Lyapunov exponent can be extracted for this position. This is due to the presence of the interface beneath them, which captures the tracers due to its undulating motion. Different positions above position B have also been tested (results not shown): we observe that when the tracers are set further above position B (i.e., further above the interface), Lyapunov exponents can be extracted again, and lead to results similar to those of position C. Our hypothesis is that the mixing is attenuated near the interface since the direction of the flow follows the motion of the interface. Thus, there is mainly a vertical shear in this area and the distance between particles at the same altitude remains similar, only the evolution of the vertical distance measured between particles matters. Figure 14 shows the Lyapunov exponents γ obtained for positions A, C, D, and E. The highest values of γ are obtained for the tracers located in position A, which are on the trajectory of a cilium and at an altitude of 0.45*L*. The values of γ corresponding to position E are smaller, which makes sense as the tracers are on the trajectory of the same cilium, but much closer to the epithelial surface. Thus, since the velocity of the cilium is smaller near its base, the mixing is weaker. Interestingly, the tracers of position D, which are in the middle of the PCL but between two cilia along the *y*-direction, give values of γ smaller that the ones of position E. This indicates that the mixing in areas which are not on the trajectory of a cilium is much weaker. Moreover, it takes also more time for the separation distance between fathers and children to increase: around 25 cycles for tracers in position D against only 10 cycles for tracers in position A. Finally, far above the mucus-PCL interface, the values obtained for γ are very small: the mixing is almost null. The trend of the curve for position E is the same as for positions A, C, and D. It is worth noticing that the same trend is observed for the Lyapunov exponents in Figure 14 and the total displaced volume of fluids in Figure 4. Indeed, the mixing in the present configuration is due to the combined action of mixing by chaotic advection and by stretching. While the major contribution for the obtained values of the Lyapunov exponents extracted comes from their initial positions (A, B, C, D, or E), the shape of the curves in Figure 14 is due to the combined action of these two phenomena. It is however reasonable to think that the regions of stronger stretching are also the regions where the chaotic mixing is the strongest. Hence, the extracted Lyapunov exponents are suitable for a qualitative measure of the mixing as ΔΦ varies. More details on flow patterns associated to peculiar phase lags can be obtained in Chateau et al. (2017).

### 4.3. Advective and diffusive time scales

The aim of this part is to compare the mixing time scales associated with chaotic advection to those associated with molecular diffusion in the PCL and in the mucus. To do so, we follow the procedure described in Ding et al. (2014) which is recalled hereafter. Note that the main difference here, compared to the work of Ding et al. (2014), is the use of two fluid layers instead of just one, which allows us to investigate different mixing behaviors between the PCL and mucus layers. First, as in section 4.1, we consider particles of different colors initially seeded at a distance *s*_0_ apart at *t* = *t*_0_. At *t* > *t*_0_, the distance between these two populations of particles has decreased by a ratio α, where 0 < α < 1. Assuming there is only fluid advection, it takes *N* cycles for the separation distance between the particles to become *s*_*N*_ = (1 − α)*s*_0_. The definition of *s*_*N*_ is thus equivalent to the one of the mixing number *m* introduced in section 4.1. If the mixing is chaotic, i.e., if the decay in particle separation distance is exponential, one gets: $s_{N}^{2}=s_{0}^{2}\exp^{-\beta N}$. Hence, the time scale associated with mixing by fluid advection is :

(20) $$\begin{array}{r} t_{mixing}^{\alpha }=T_{osc}\;*\;N=\frac{2\pi N}{\omega }=-\frac{4\pi \log\left(1-\alpha \right)}{\beta \omega } \end{array}$$

where ω is the cilia beating frequency. From a molecular diffusion standpoint, particles moving on a distance α*s*_0_ by molecular diffusion with a diffusivity coefficient *D* would have the following characteristic time:

(21) $$\begin{array}{r} t_{diffusion}=\frac{{\left(\alpha s_{0}\right)}^{2}}{D} \end{array}$$

By equating the two time scales, one gets:

(22) $$\begin{array}{r} \omega =\frac{4\pi \log\left(1-\alpha \right)}{{\left(\alpha s_{0}\right)}^{2}\beta }D \end{array}$$

**Figure 15:**
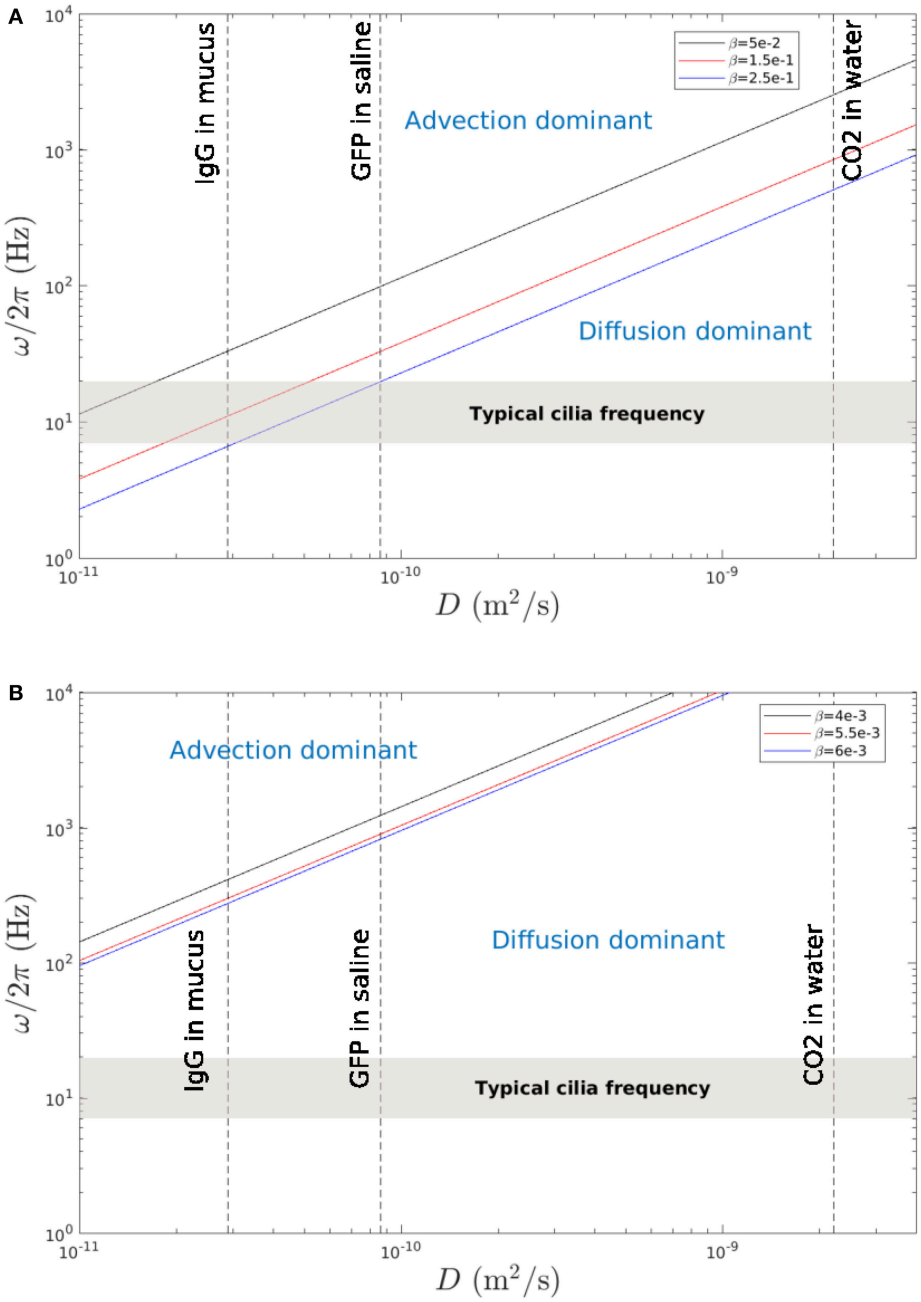
Cilia beating frequency ω/2π as a function of the diffusivity coefficient *D*. The lines show when the time scale due to mixing by molecular diffusion is equal to the time scale due to mixing by fluid advection. **(A)** Results obtained in area 1 (PCL region) for typical mixing rates β = 0.05, 0.15, and 0.25. The gray region represents the typical beating frequencies of cilia. The dashed line indicate the diffusion coefficients for human IgG in mucus (Saltzman et al., 1994), GFP (Green Fluorescent Protein) in aqueous saline (Swaminathan et al., 1997), and CO_2_ in water (Fridlyand et al., 1996). The phase diagram obtained is similar to the one of Ding et al. (2014). **(B)** Results obtained in area 2 (above the PCL-mucus interface) for representative mixing rates β = 4.10^−3^, 5.10^−3^, and 6.10^−3^.

Thus, for given α, *s*_0_ and β, Equation (22) gives a linear relationship between ω and *D* which allows to compare in the parameter space (*D*, ω) the regions where the mixing is dominated by advection or by molecular diffusion. In order to compare our results to the ones of Ding et al. (2014), the same values of α = 0.9 and *s*_0_ = *L* = 10 μm are used. Figure 15A,B show the results obtained in the PCL (area 1) and in the mucus (area 2) respectively. One can see in Figure 15A that there is a region compatible with typical cilia beating frequency where mixing by fluid advection is dominant. This is in accordance with the results found by Ding et al. (2014) who obtained similar mixing rates in a single layer of fluid. Note that in Ding et al. (2014), as only one phase was modeled, only two populations of tracers were considered, which filled the whole computational domain. No distinction in Ding et al. (2014) was made between regions of strong mixing (around the cilia), and regions of weaker mixing (far above the cilia). On the contrary, Figure 15B shows that above the PCL-mucus interface, the mixing is dominated by molecular diffusion. Hence, it shows that drugs deposited onto the mucus layer can only reach the PCL via molecular diffusion. This can be confirmed by doing a simple calculus: according to Morgan et al. (2004), the mucus velocity is around $V_{\text{mucus}}=1.72.10^{-4}$ m.s^−1^. Assuming that there are no bifurcations in the airways, so that the mucus is transported in the same direction on a total length of around 20 cm, and assuming that its velocity *V*_mucus_ remains constant, one gets that it takes around 20 h for the mucus to be expelled. This time has to be compared with the time taken by particles for reaching the PCL layer by molecular diffusion: assuming that the layer of mucus has a thickness *L*_*mucus*_ = 70 μm, the time for a particle to diffuse over this distance can be approximated using Equation (21): $t\approx {\left(\alpha s_{0}\right)}^{2}/D=L_{mucus}^{2}/D$. Using a diffusion coefficient *D* = 2.9.10^−11^ m^2^.s^−1^, corresponding to human immunoglobulin G (IgG) in mucus (Saltzman et al., 1994), one gets a value of 169 s for the IgG to reach the PCL-mucus interface. These results show that drugs injected by nasal sprays and deposited onto the mucus layer may always reach the PCL area through molecular diffusion. There, the chaotic advection will further increase the mixing to bring drugs near the epithelium. However, drugs composed of large molecules will have smaller diffusion coefficients, and might not reach the PCL in time (for instance, for a value of diffusion coefficient of the order of 10^−14^, it will take around 136 h to reach the PCL). However, note that the conclusions drawn here result from several hypothesis, which may limit the generality of our simplified model of MCC. Other phenomena, such as chemical reactions, osmosis, or unusual mucus properties associated to peculiar pulmonary diseases might occur and should be taken into account for a deeper understanding of the balance between advective and diffusive mixing.

## 5. Conclusion and perspectives

By using a coupled lattice-Boltzmann/Immersed Boundary solver, the transport and mixing induced by beating cilia were studied in the context of MCC. Thanks to this numerical approach, a stable two-phase system (mucus-PCL), allowing the introduction of a viscosity ratio, can be studied. The mucus-PCL interface is also naturally captured. Due to the local nature of the LBM, the parallelization is straightforward, allowing the simulations of large domains.

A detailed study of the transport induced by antiplectic and symplectic MCW has been performed, and the results showed that antiplectic MCW with large wavelengths (i.e., ΔΦ < π/4) are more able to transport the mucus. A displacement ratio has also been introduced to quantify the capacity of a system to transport particles for a given power input. The configuration corresponding to an antiplectic MCW with ΔΦ = π/6 has been found to be the most energetically efficient. On the contrary, symplectic MCW with large wavelengths result in a very poor transport, due to the displacement of the mucus-PCL interface above the cilia tips during their stroke phase.

The mixing capabilities of the system have also been studied in three distinct areas. The results showed that the mixing is chaotic in both the PCL region and above the PCL-mucus interface. The stronger mixing is obtained in the PCL region where only a few beating cycles are required to obtain a converged state of mixing. On the contrary, far above the interface, the mixing is almost null. The calculation of Lyapunov exponents in specific locations of the domain has also shown that the mixing is stronger when a cilium passes through the area of measurements, and especially around the cilia tips because of their “whip-like” motion. On the contrary, between two cilia along the *y*-direction, the mixing takes more time and is weaker. At the interface, particles are trapped and consequently follow the undulating motion of the mucus-PCL interface. Two time scales can be defined, one associated with advective mixing and the other one with diffusive mixing. The results showed that in the mucus, the mixing is always dominated by diffusion. Regions in the ω-*D* phase diagram where mixing in the PCL is dominated by advection also exist. These results show that drugs deposited onto the mucus layer can only reach the PCL layer via molecular diffusion. The two-layer character of the MCC allows a strong chaotic mixing in the PCL while trapping the particles inside thanks to the presence of a viscous layer of mucus. Above, the mixing is also chaotic, but at a much lower rate, which allows the mucus to be transported straightforwardly.

Future efforts toward more realistic simulations of the MCC include:

- The implementation of a non Newtonian rheological behavior for the mucus based on the experiments of Lafforgue et al. (2017);
- to introduce a porous epithelial surface, to capture ions transfer using the model developed by Pepona and Favier (2016);
- the implementation of a more realistic 3D beating pattern for the cilia (Gheber and Priel, 1997).

## Author contributions

SC worked on the numerical framework, designed the analysis, and drafted the manuscript. UD developed the algorithm, helped in data analysis, provided significant feedback on the computation of the Lyapunov exponents, and reviewed the manuscript. SP developed the algorithm, helped in data analysis, and reviewed the manuscript. JF developed the algorithm, helped in data analysis, and reviewed the manuscript.

### Conflict of interest statement

The authors declare that the research was conducted in the absence of any commercial or financial relationships that could be construed as a potential conflict of interest.
